# Transmission, Reflection and Dissipation of Microwaves in Magnetic Composites with Nanocrystalline Finemet-Type Flakes

**DOI:** 10.3390/ma14133499

**Published:** 2021-06-23

**Authors:** Anatoly B. Rinkevich, Dmitry V. Perov, Yuriy I. Ryabkov

**Affiliations:** 1 M.N. Miheev Institute of Metal Physics UB RAS, Sofia Kovalevskaya St., 18, Ekaterinburg 620108, Russia; peroff@imp.uran.ru; 2 Institute of Chemistry UB RAS, Pervomayskaya St., 48, Syktyvkar 167000, Russia; ryabkov-yi@chemi.komisc.ru

**Keywords:** magnetic composites, microwaves, absorption, transmission and reflection coefficients

## Abstract

The microwave properties of a composite material containing flakes of finemet-type nanocrystalline alloy placed in the epoxy matrix have been investigated. Two compositions have been studied: with 15% and 30% flakes. Frequency dependences of transmission and reflection coefficients are measured in the frequency range from 12 to 38 GHz. The dielectric permittivity and magnetic permeability are obtained, and the microwave losses are calculated. The dependences of transmission and reflection coefficients have been drawn as functions of wave frequency and thickness of the composite material, taking into account the frequency dependences of permittivity and permeability. The regions of maximal and minimal microwave absorption have been defined. The influence of wave interference on the frequency dependence of microwave absorption is studied.

## 1. Introduction

The study of nanocomposites consisting of metallic nanoparticles and polymer matrices has become a significant active field due to their physical properties attractive for applications [1]. There is large variety of mixing formulas allowing determine the permittivity of a composite media if concentrations and permittivities of its components are assumed known [2]. The microwave magnetic properties of composites and the mixing rules are briefly reviewed in [3], and the laws governing the magnetic frequency dispersion in magnetic composites are discussed. The composites containing magnetic metallic particles are regarded as electromagnetic wave absorbers and as materials for other devices such as microwave antennas, materials for mobile communications, etc. A method of studying microwave magnetic properties of metal particles based on swept frequency measurements under magnetic bias in a coaxial line is developed [4]. Polymer media, ceramics and other dielectrics with high-quality factors are often be chosen as matrices for composites [5].

The best microwave absorbing materials should fulfil such requirements as lightweight, more effective and broader bandwidth absorption. The types of fillers and polymer matrices, as well as the number of layers and thickness, have to be chosen to improve the absorbing capability [6]. The frequency dispersion of flake-shaped and spherical-like Fe_16_Ni_82_Mo_2_ alloy particles has been studied [7]. The composite that contains Fe-50 wt% Ni alloy particles with spherical form has been prepared to improve the microwave absorbing properties in the range of 1–4 GHz [8]. The complex permittivity, complex permeability and reflection loss of the microwave absorbing material are also studied. Improved electromagnetic shielding and absorption properties are obtained for a polymer–metal composite based on polyvinylidene fluoride dispersed with varying concentration of nanocrystalline iron [9]. The possibilities of improving the shielding and absorptive properties of carbon-based admixtures, nickel powder, iron powder, ferrites, magnetite and other materials from the pulsed, high power microwave irradiation have been reviewed in [10].

The dielectric and magnetic losses in the granular structures constituted by ferromagnetic nanoparticles (Co, Fe, B) in an insulating amorphous SiO_2_ matrix are investigated at microwave frequencies. The magnetic losses are caused mainly by the fast spin-polarized relaxation mechanism [11]. The microwave refraction coefficient of a composite consisting of Fe-Si-Nb-Cu-B alloy flakes placed into an epoxy resin matrix is investigated. It has been shown that the material under consideration behaves as a dielectric at direct current (DC) and as a lossy dielectric at microwave frequencies in the absence of a magnetic field. Near the field of ferromagnetic resonance, the real and imaginary parts of the complex refraction coefficient are of the same order as in a conductive medium [12]. Systematic permeability measurements of magnetically structured granular systems using the transmission/reflection waveguide method are carried out. The effective complex permeability is measured in the frequency range of 0.01–10 GHz [13]. The distribution of the ferromagnetic Ni in the ceramic matrix of ZrO_2_ is studied [14]. The gradient nanocomposite films are promising for better matching between air and metal in the microwave shielding problem.

Due to high magnetic permeability, the finemet-type alloys are regarded as suitable materials for radio- and microwave engineering. The frequency response of magnetic cores produced from pulverized FeNbBSiCu-based nanophase alloy ribbon is studied [15]. The measurements of the magnetic properties of powdered Fe_73.5_Cu_1_Nb_3_Si_13.5_B_9_ nanostructured alloy have been carried out in the frequency range 0.2–10.2 GHz [16]. Optimization of magnetic properties and magnetic anisotropy of thin, soft magnetic films of finemet alloys is accomplished [17]. The 100–200 nm films are found to be appropriate materials for sensor and actuator devices. Coupling between microstructure and the magnetic properties of FeNbBSiCu alloys is discussed systematically in the paper [18]. The best soft magnetic performance can be obtained when the average nanocrystal size is 16 nm. The FeNbBSiCu thin films have been deposited using RF sputtering [19]. The magnetic field microsensors based on the magneto-impedance effect have been fabricated by stacking up finemet/copper/finemet films. Thus, the thin film and composite materials with finemet alloys are applicable in many microelectronic devices and magnetic field sensors.

To build up the composite materials, which have optimal properties, it is necessary to elaborate on the methods of calculation of microwave dielectric and magnetic losses. The complexity of this problem lies, among other factors, in the fact that the shape, the dimensions and the spatial orientation of ferromagnetic particles have to be considered. The problems arising in the calculation of the dynamic magnetic permeability of composites have been discussed in [20,21,22]. A possible method of introduction of the magnetic permeability tensor based on the Maxwell–Garnet model is presented in the paper [20]. The difference between a magnetic field inside of a ferromagnetic particle and a given magnetic field outside it can be defined using the effective demagnetizing tensor, which depends on the portion of the ferromagnetic phase in the composite [21,22].

The calculation of magnetic losses is important in order to estimate microwave absorption. The computation of the effective magnetic permeability is worked out in this paper for composite materials containing ferromagnetic particles. The calculation is performed on the frequency dependences of the microwave transmission and reflection coefficients. The measurements of these coefficients are carried out for the composite material with the flakes made of finemet-like alloy in the frequency range from 12 to 38 GHz. The performed calculation allows one to choose the optimal conditions, namely the thickness of the plate and the wave frequency, for maximal or, in opposite, minimal reflection coefficient and dissipated power inside the composite. The comparison is drawn between the experimental data on microwave dissipation and computed ones. The role of wave interference inside the composite plate is clarified.

## 2. Materials and Methods

Particles of Fe-Si-Nb-Cu-B alloy in the form of flakes have been used for the preparation of the composite. The chemical analysis is carried out with the X-ray fluorescence analyzer Horiba 500 as well as with the method of atomic emission spectroscopy with the inductive-coupled plasma. The elemental composition of the flakes is as follows: Fe—80.1%; Si—8.5%; Nb—8.4%; Cu—1.1%; B—1.2%; Cr—0.2%; Mn—0.1%; Ni—0.1%; Co—0.3%.

**Figure 1:**
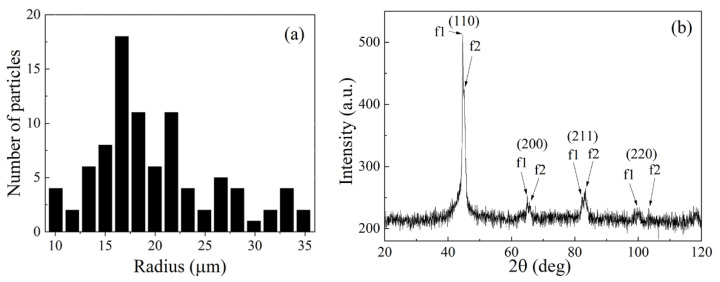
The distribution of mean radiuses of flakes (**a**); the X-ray diffraction image of the composite medium (**b**).

It is a nanocrystalline finemet-like alloy with high magnetic permeability. The distribution of the mean radii of the flakes is shown in Figure 1a. The mean radius, which is the mean distance from the center of mass to the border, is 20.1 μm. The maximal Martin diameter, which is the maximal length of a line fit into a particle, is 51 μm on average, and the minimal Martin diameter is 27 μm. The ratio of these diameters equals 1.9, which characterizes the relation between the lateral sizes.

The composite material is prepared by mechanical mixing of flake particles in an epoxy oligomer to matrix polymerization. The epoxy matrix is chosen because of its moderate dielectric permittivity as well as the simplicity of preparation of the composite. After mechanical mixing, the treatment in an ultrasonic bath is performed. Further, the liquid mixture of epoxy and particles is sealed in the metallic mounts, which cavities have similar dimensions as the lateral dimensions of the rectangular waveguides in which the microwave measurements are carried out. Hardening of the mixture lasts several hours. Two sequences of composite samples have been prepared with 15 wt.% and 30 wt.% of flakes. The X-ray phase analysis with a “Pananalytical” spectrometer shows that the main phases are two phases of *bcc* lattice of *α*-Fe type (f1 and f2), distinguished only by the lattice parameters, which equal to 2.871 Å and 2.841 Å. This difference can be explained as follows. Because the significant amounts of Si and Nb are present in the finemet-like alloy, two phases are formed during the crystallization from a homogeneous melt; these are *α*-Fe–Nb and *α*-Fe–Si. The atomic radii of these solid solutions differ from the atomic radius of Fe. The phases cause the splitting of the main picks of an X-ray diffraction pattern into the doublets. The X-ray diffraction image for the sample with 15% flakes is shown in Figure 1b.

**Figure 2:**
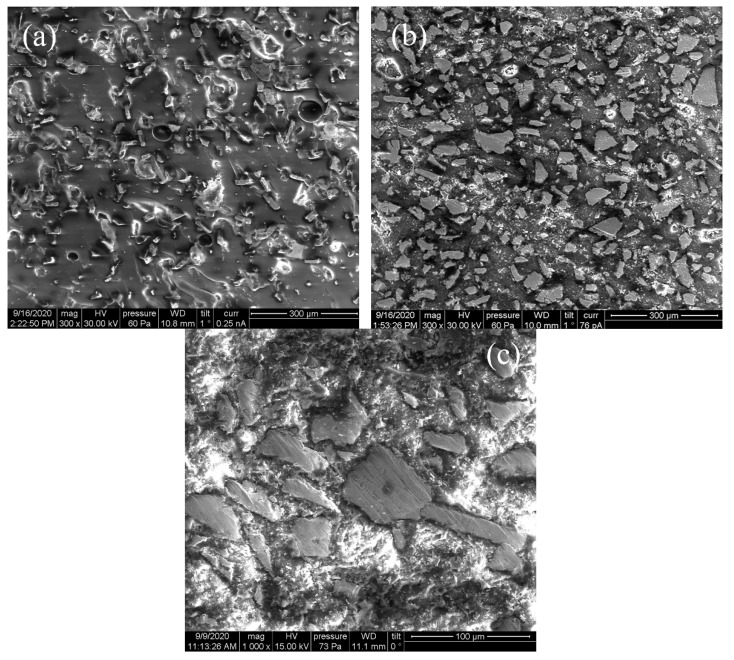
The structure of the composite medium with 15% flakes: chip (**a**); its top surface (**b**); the structure of the composite medium with 30% flakes obtained from its top surface (**c**).

The structure of the composite is studied with the Vega3 from Tescan, Brno, Czech republic electron microscope at an accelerating voltage of 30 kV. The structure of the composite with 15% flakes is shown, and the samples are prepared from the chip, i.e., from the inner part of the sample (Figure 2a) and the top surface (Figure 2b). It should be noted that the particles in the inner part of the sample are oriented stochastically, but on the top surface, there is a preferred orientation of the particles in parallel to the surface. The preferred orientation of flakes is seen also for the top view of the composite with 30% flakes (Figure 2c). The preferred orientation in the top part of the sample is formed during the hardening process because of the influence of surface tension forces. From the microscopy data, one can conclude that approximately 20% of flakes have preferable orientation in parallel to the surface, and 80% of flakes are oriented stochastically. Let us also point out that electric contact between the flakes is absent, so the DC conductivity is negligible.

**Figure 3:**
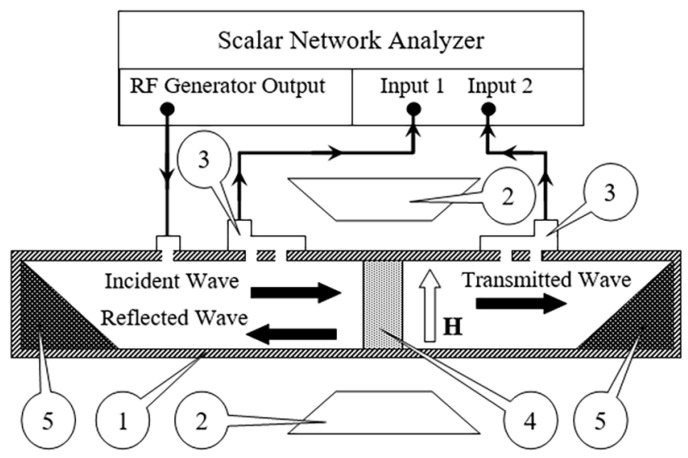
The scheme of microwave measurements: 1—the waveguide; 2—the electromagnet; 3—the directional couplers; 4—the sample; 5—the microwave absorber.

The microwave measurements are performed at frequencies from 12 to 38 GHz according to the method described in [23]. The scheme of the experiment is shown in Figure 3. The sample is placed into a rectangular waveguide *1* to completely overlap its cross-section. The thickness of the sample is from 1.5 to 2 mm. The waveguide operates at TE_10_ mode, and its dimensions are defined by the frequency range: 16 mm × 8 mm for 12–17 GHz (WR-62); 11.5 mm × 5 mm for 17–26 GHz (WR-42); 7.2 mm × 3.4 mm for 27–38 GHz (WR-28). The wave impinges upon the surface of the sample normally. The measurements are carried out with the scalar network analyzer. The amplitudes of transmitted and reflected waves are measured with directional couplers *3*. The modules of transmission *T* and reflection *R* coefficients have been measured as well as their frequency dependences. The measurements of the coefficients are used to determine the complex dielectric permittivity $\dot{\varepsilon }$.

Let us shortly describe the procedure of measurements and calculations of the complex dielectric permittivity. The complex transmission $\dot{T}$ and reflection $\dot{R}$ coefficients can be calculated via the formulas [24,25]:

(1) $$\dot{T}=\frac{2Z_{1}{\dot{Z}}_{2}}{2Z_{1}{\dot{Z}}_{2}\cos\left({\dot{k}}_{2}d_{2}\right)+i\left(Z_{1}^{2}+{\dot{Z}}_{2}^{2}\right)\sin\left({\dot{k}}_{2}d_{2}\right)}$$

(2) $$\dot{R}=\frac{i\left({\dot{Z}}_{2}^{2}-Z_{1}^{2}\right)\sin\left({\dot{k}}_{2}d_{2}\right)}{2Z_{1}{\dot{Z}}_{2}\cos\left({\dot{k}}_{2}d_{2}\right)+i\left(Z_{1}^{2}+{\dot{Z}}_{2}^{2}\right)\sin\left({\dot{k}}_{2}d_{2}\right)}$$

In Equations (1) and (2), the medium “1” is either the inner space of the waveguide or free space. The medium “2” is the sample, i.e., imperfect dielectric ferromagnet with the thickness *d*_2_.

The complex impedance ${\dot{Z}}_{2}$ for this medium can be written as follows:

(3) Z˙2=ReZ˙2+iImZ˙2=μ0μ˙ε0ε˙=μ0|μ˙|ε0|ε˙|⋅exp[−i(argμ˙−argε˙)2]

$$\left|{\dot{Z}}_{2}\right|=\sqrt{\frac{\mu_{0}\left|\dot{\mu }\right|}{\varepsilon_{0}\left|\dot{\varepsilon }\right|}}$$

ReZ˙2=μ0|μ˙|ε0|ε˙|⋅cos[(argμ˙−argε˙)2]ImZ˙2=μ0|μ˙|ε0|ε˙|⋅sin[−(argμ˙−argε˙)2]

Re(Z˙22)=μ0|μ˙|ε0|ε˙|⋅cos(argμ˙−argε˙)

The impedance of the space “1” for the waveguide is

(4) $$Z_{1}=\sqrt{\frac{\mu_{0}}{\varepsilon_{0}}}\frac{1}{\sqrt{1-{\left(\frac{\pi c}{\omega a}\right)}^{2}}}$$

and the same for the free space

$$Z_{1}=\sqrt{\frac{\mu_{0}}{\varepsilon_{0}}}$$

where $\mu_{0}$ and $\varepsilon_{0}$ are the magnetic permeability and dielectric permittivity of vacuum, $c=\frac{1}{\sqrt{\varepsilon_{0}\mu_{0}}}$ is the speed of light, *a* is the greater size of cross section of the rectangular waveguide, and ω=2πf is the cyclic frequency.

The complex constitutive parameters of the sample, namely, the magnetic permeability $\dot{\mu }=\mu^{\prime }-i\mu^{\prime \prime }$ and dielectric permittivity $\dot{\varepsilon }=\varepsilon^{\prime }-i\varepsilon^{\prime \prime }$ enter the Equation (3). The designations $\mu^{\prime }$, $\varepsilon^{\prime }$ and $\mu^{\prime \prime }$, $\varepsilon^{\prime \prime }$ correspond, respectively, to the real and imaginary parts of the permeability and permittivity. Similar marking for the components of complex values will be used throughout the article. The complex wavenumber ${\dot{k}}_{2}=k_{2}^{\prime }-ik_{2}^{\prime \prime }$ for an imperfect dielectric ferromagnet in Equations (1) and (2) is calculated in the following manner. For TE_10_ mode of a rectangular waveguide, the wavenumber’s components are equal to

(5) $$\begin{array}{c} k_{2}^{\prime }=\sqrt{\frac{\sqrt{{ℜ}^{4}+{ℑ}^{4}}+{ℜ}^{2}}{2}},\\ k_{2}^{\prime \prime }=\sqrt{\frac{\sqrt{{ℜ}^{4}+{ℑ}^{4}}-{ℜ}^{2}}{2}}, \end{array}$$

where $ℜ=\sqrt{{\left(\frac{\omega }{c}\right)}^{2}\left(\varepsilon^{\prime }\mu^{\prime }-\varepsilon^{\prime \prime }\mu^{\prime \prime }\right)-{\left(\frac{\pi }{a}\right)}^{2}}$ and $ℑ=\frac{\omega }{c}\sqrt{{\left(\varepsilon^{\prime \prime }\mu^{\prime }+\varepsilon^{\prime }\mu^{\prime \prime }\right)}^{}}$, and for the free space

$$\begin{array}{c} k_{2}^{\prime }=\frac{\omega }{c}\sqrt{\frac{\left|\dot{\varepsilon }\right|\left|\dot{\mu }\right|+\varepsilon^{\prime }\mu^{\prime }-\varepsilon^{\prime \prime }\mu^{\prime \prime }}{2}},\\ k_{2}^{\prime \prime }=\frac{\omega }{c}\sqrt{\frac{\left|\dot{\varepsilon }\right|\left|\dot{\mu }\right|-\varepsilon^{\prime }\mu^{\prime }+\varepsilon^{\prime \prime }\mu^{\prime \prime }}{2}}. \end{array}$$

Additionally, the complex impedance ${\dot{Z}}_{2}$, which is defined by Equation (3) for an unbounded medium, for the TE_10_ mode of the waveguide takes the view:

$${\dot{Z}}_{2}=\frac{\omega \mu_{0}\dot{\mu }}{{\dot{k}}_{2}}$$

where the wavenumber ${\dot{k}}_{2}$ is determined by equation for free space.

The power transmission $T_{P}$ and reflection $R_{P}$ coefficients are formally given by the equations

(6) $$\begin{array}{c} T_{P}=\dot{T}\cdot T^{*}=\\ =\frac{4Z_{1}^{2}{Z_{2}^{*}}^{2}}{\left[2Z_{1}{\dot{Z}}_{2}\cos\left({\dot{k}}_{2}d_{2}\right)+i\left(Z_{1}^{2}+{\dot{Z}}_{2}^{2}\right)\sin\left({\dot{k}}_{2}d_{2}\right)\right]\left[2Z_{1}Z_{2}^{*}\cos\left(k_{2}^{*}d_{2}\right)-i\left(Z_{1}^{2}+{Z_{2}^{*}}^{2}\right)\sin\left(k_{2}^{*}d_{2}\right)\right]} \end{array}$$

(7) $$\begin{array}{c} R_{P}=\dot{R}\cdot R^{*}=\\ =\frac{\left({\dot{Z}}_{2}^{2}-Z_{1}^{2}\right)\left({Z_{2}^{*}}^{2}-Z_{1}^{2}\right)\sin\left({\dot{k}}_{2}d_{2}\right)\sin\left(k_{2}^{*}d_{2}\right)}{\left[2Z_{1}{\dot{Z}}_{2}\cos\left({\dot{k}}_{2}d_{2}\right)+i\left(Z_{1}^{2}+{\dot{Z}}_{2}^{2}\right)\sin\left({\dot{k}}_{2}d_{2}\right)\right]\left[2Z_{1}Z_{2}^{*}\cos\left(k_{2}^{*}d_{2}\right)-i\left(Z_{1}^{2}+{Z_{2}^{*}}^{2}\right)\sin\left(k_{2}^{*}d_{2}\right)\right]} \end{array}$$

where the asterisk means the complex conjugation.

If one knows the coefficients from Equations (6) and (7), the dissipation *D* can be calculated, which presents the portion of the microwave power dissipated inside the sample:

(8) $$D=1-T_{P}-R_{P}$$

Dissipation of the microwave power occurs for the following reasons: absorption in the sample, scattering on the inner heterogeneities and a transformation into the evanescent modes of the waveguide at the boundaries of the sample.

Let us first accept the magnetic permeability as a known value and discuss the procedure how to extract the complex dielectric permittivity from the measured frequency dependences of transmission and reflection coefficients moduli [23]. Denote the measured experimentally frequency dependence of transmission coefficient modulus as $\left|\tilde{T}(\omega \dot{,}\dot{\varepsilon },\dot{\mu })\right|$ and reflection coefficient modulus as $\left|\tilde{R}(\omega \dot{,}\dot{\varepsilon },\dot{\mu })\right|$. Let us write the difference between calculated |T| and measured $\left|\tilde{T}\right|$ values of the transmission coefficient modulus as $\Delta_{T}=\left|{\tilde{T}}_{p}(\omega ,\dot{\varepsilon },\dot{\mu })\right|-\left|T_{p}(\omega ,\dot{\varepsilon },\dot{\mu })\right|$, and in a similar manner, for the reflection coefficient, $\Delta_{R}=\left|{\tilde{R}}_{p}(\omega ,\dot{\varepsilon },\dot{\mu })\right|-\left|R_{p}(\omega ,\dot{\varepsilon },\dot{\mu })\right|$. Here, the complex dielectric permittivity $\dot{\varepsilon }$ is an unknown value. To find it, one chooses $\dot{\varepsilon }$ so that the full difference

(9) $$\Delta =\underset{\dot{\varepsilon }}{\min}\left[\sqrt{{\left(\Delta_{R}\left(\omega ,\dot{\varepsilon },\dot{\mu }\right)\right)}^{2}+{\left(\Delta_{T}\left(\omega ,\dot{\varepsilon },\dot{\mu }\right)\right)}^{2}}\right]$$

has a minimum. The obtained value of $\dot{\varepsilon }$ are accepted as an estimation of the dielectric permittivity. To fulfil the minimization procedure, the frequency range should be chosen in which the amplitude-frequency characteristics of *T* and *R* coefficients are measured. The full working frequency range of the waveguide can be initially accepted.

If for none of the frequency belonging to this range, the difference between |T| and $\left|\tilde{T}\right|$, as well as between |R| and $\left|\tilde{R}\right|$, does not exceed a preassigned value (this assigned value defines the accuracy of estimation of $\dot{\varepsilon }$), then one can regard the obtained value of $\dot{\varepsilon }$ is constant within the chosen frequency range. The latter is true if the frequency dispersion of $\dot{\varepsilon }$ is weak. If, however, the difference exceeds the preassigned value, determination of $\dot{\varepsilon }$ and the minimization procedure repeats using the sliding frequency window. The minimization procedure (9) is performed at every position of the sliding window, and the frequency dependence of $\dot{\varepsilon }(\omega )$ is obtained as a result. From the obtained $\dot{\varepsilon }$, the microwave conductivity can be calculated: $\sigma =\omega \varepsilon_{0}\varepsilon^{\prime \prime }$.

## 3. Results

**Figure 4:**
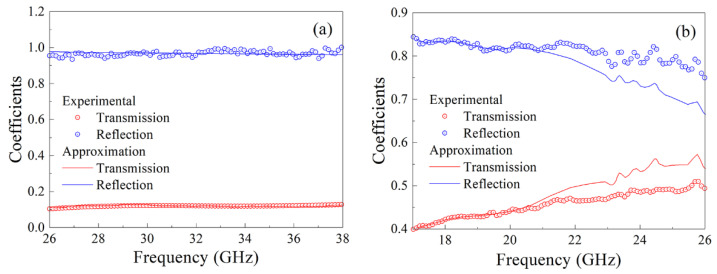
Frequency dependences of transmission and reflection coefficients: for the composite with 30% flakes in the frequency range from 26 to 38 GHz (**a**); for the composite with 15% flakes in the frequency range from 17 to 26 GHz (**b**).

The frequency dependences of transmission and reflection coefficient modules have been measured for the composite samples with 15% and 30% ferromagnetic particles. The measurements are carried out within three frequency ranges: from 12 to 17 GHz, from 17 to 26 GHz, from 26 to 38 GHz, with the corresponding waveguide in every frequency range. The results of measurements are presented partially in Figure 4. It is found that for the composite with 30% flakes in the frequency range from 26 to 38 GHz, the dependences calculated with one optimally chosen $\dot{\varepsilon }$ value fairly good approximate the measured dependences, see Figure 4a. For the composite with 15% flakes, the frequency dependences of transmission and reflection coefficients moduli have an essential difference between the measured and calculated dependences at frequencies above 20.5 GHz. The method with the sliding window is used in this case, and the $\dot{\varepsilon }(\omega )$ dependence has been obtained, which is presented in the next section. Depending on the frequency dispersion of $\dot{\varepsilon }(\omega )$ function, the width of the sliding window is chosen from 0.4 to 4 GHz. At that, the difference between approximated and measured dependences of *T* and *R* does not exceed 0.02.

**Figure 5:**
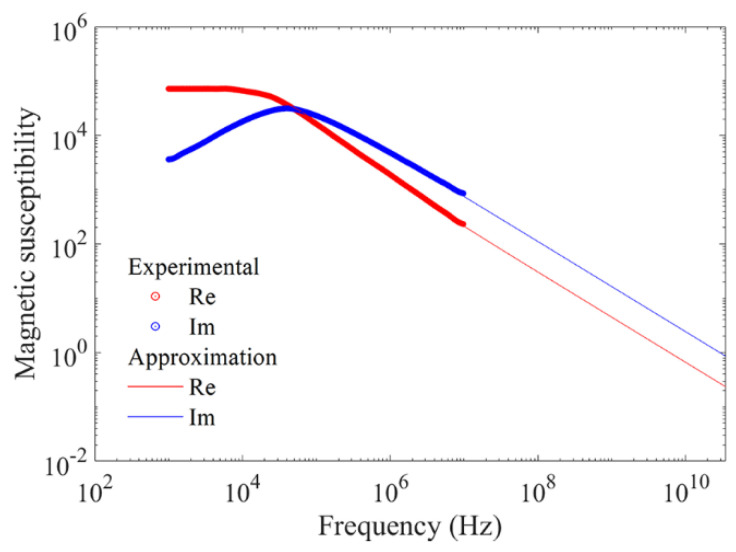
A comparison between measured and approximated frequency dependences of the real and the imaginary parts of magnetic permeability of Finemet alloy.

Let us present the data on magnetic permeability and dielectric permittivity of the composite samples. The method of how the dielectric permittivity has been obtained is described above. For magnetic permeability, the problem lies in the fact that there is no measured frequency dependence of magnetic permeability for so high frequencies in literature. Let us, at first, obtain the approximation of frequency dependency of the material from which the flakes are produced and then calculate the permeability of the composite material. The frequency dependence of complex magnetic permeability of Hitachi Finemet alloys in the frequency range from 1 kHz to 10 MHz is presented in [26], see Figure 5. The values of magnetic permeability for the material of flakes at frequency *f* = 10 GHz are given in the patent [27]. In Figure 5, besides the experimental data on permeability, the approximation using the Cole–Cole formula [28] is also shown. This formula represents a typical view of the frequency dependence

(10) $$\dot{\chi }\left(\omega \right)=\chi^{\prime }\left(\omega \right)-i\chi^{\prime \prime }\left(\omega \right)=\chi_{\infty }+\frac{4\pi (\chi_{0}-\chi_{\infty })}{1+{\left(i\omega \tau \right)}^{1-\alpha }}$$

In Equation (10), $\chi_{0}$ is the permeability at *ω*→0, $\chi_{\infty }$ is the permeability at ω→∞, *τ* is the mean relaxation time of the magnetic moment, which is an adjustable parameter, and α is the parameter chosen for better approximation, 0 < *α* < 1.

From the view of dependences shown in Figure 5, let us accept $\chi_{\infty }$ = 0 because, with the increase in frequency, the monotonic and strong decrease in permeability is fixed. The best results of approximation have been obtained with $\chi_{0}$ = 1.78 · 10^3^, τ = 3.84 · 10^−6^ s, α = 0.17. At these values of the approximation parameters at frequency *f* = 10 GHz Re(μ˙) ≈ 1.2, in accordance with data [27], and at frequency *f* = 30 GHz Re(μ˙) ≈ 1.08. Equation (10) permits calculating the magnetic susceptibility and permeability for the material of flakes in a wide frequency range. After that, it is necessary to calculate the magnetic permeability of the composite.

The calculation of magnetic permeability is carried out for the ensemble of ferromagnetic particles flakes-type composed of 1600 randomly oriented flakes and 400 flakes parallel to the top surface of the sample.

The complex effective magnetic permeability of the composite ${\dot{\mu }}_{eff}$ is calculated using the formula

(11) $${\dot{\mu }}_{eff}=\langle {\dot{\mu }}_{}^{m}\left(\Theta \right)\rangle$$

where index *m* means belonging to a separate ferromagnetic particle, and the angle brackets mean averaging over the ensemble, taking into consideration the angle **Θ** of the orientation of every flake. The spatial orientation of a flake can be characterized by the direction of the normal to its flat surface. The orientation of a ferromagnetic particle is given by the vector $\Theta =\left(\begin{array}{ccc} \alpha & \beta & \gamma \end{array}\right)$, where *α*, *β* and *γ* are the elements of the independent sets of random numbers with the uniform laws of distribution and belonging to the following intervals: α∈[−π; π], β∈[−π; π] and γ∈[−π; π].

In computer modelling of ${\dot{\mu }}_{eff}$, the discrete sets of numbers $\alpha_{p}$, $\beta_{p}$ and $\gamma_{p}$ are formed by a random-number generator; they are combined into the corresponding set of the vectors $\Theta_{p}=\left(\begin{array}{ccc} \alpha_{p} & \beta_{p} & \gamma_{p} \end{array}\right)$. These vector sets specify an ensemble of the randomly oriented flakes. Take into consideration that some part of ferromagnetic particles inside the composite material is oriented in a definite manner, namely, the normal vector $n=\left(\begin{array}{ccc} 0 & 1 & 0 \end{array}\right)$ is directed along the *y-*axis; that is, these flakes lie in the top plane of the sample. This orientation is defined by the vector $\Theta_{0}$. If there are *L*_1_ randomly oriented flakes and *L*_2_ flakes with orientation $\Theta_{0}$, then Equation (11) can be rewritten in the following way:

(12) $${\dot{\mu }}_{eff}=\frac{L_{2}{\dot{\mu }}_{}^{m}\left(\Theta_{0}\right)+\sum_{p=1}^{L_{1}}{\dot{\mu }}_{}^{m}\left(\Theta_{p}\right)}{L_{1}+L_{2}}$$

In the case under consideration, we accept *L*_1_ = 1600 and *L*_2_ = 400. Equation (12) assumes that particles interact only with the external field that is correct only for low concentrations of particles. The ensemble averaging of Equations (11) and (12) over orientations can be performed using the formulas shown in the monograph [2]. Specifically, if there are the particles oriented with the tensor of demagnetizing factors $\overset{↔}{N}=1$ and $\mathrm{tr}\left(\overset{↔}{N}\right)=1$, then the tensor of magnetic permeability ${\overset{↔}{\mu }}^{m}$ of the composite medium takes the form

(13) $$\begin{array}{c} {\overset{↔}{\mu }}^{m}=\left(\begin{array}{ccc} \mu_{xx}^{m} & 0 & 0\\ 0 & \mu_{yy}^{m} & 0\\ 0 & 0 & \mu_{zz}^{m} \end{array}\right),\\ \mu_{ii}^{m}=1+\theta_{v}\frac{\mu -1}{1+\left(1-\theta_{v}\right)N_{ii}\left(\mu -1\right)}, \end{array}$$

where *μ* is the magnetic permeability of an isotropic magnetic medium that satisfies the formula $\dot{\mu }\left(\omega \right)=1+\theta_{v}\dot{\chi }\left(\omega \right)$. In Equation (13) and further, the summation is realized by the repeating indices. If the particles of the same sort are oriented randomly, then, according to [2], it is possible to introduce a scalar effective permeability of the composite medium the following view

(14) $$\mu_{eff}=1+\frac{\frac{\theta_{v}}{3}\cdot \frac{\mu -1}{1+N_{ii}\left(\mu -1\right)}}{1-\frac{\theta_{v}}{3}\cdot \frac{N_{ii}{\left(\mu -1\right)}^{}}{1+N_{ii}\left(\mu -1\right)}}$$

As soon as it can be assumed for flakes that $\overset{↔}{N}=\delta_{i2}\delta_{2j}$, where $\delta_{ij}$ is the Kronecker delta function, Equation (14) can be transformed as

(15) $$\mu_{eff}=1+\theta_{v}\left(\mu -1\right)\frac{2\mu +1}{\left(3-\theta_{v}\right)\mu +\theta_{v}}$$

**Figure 6:**
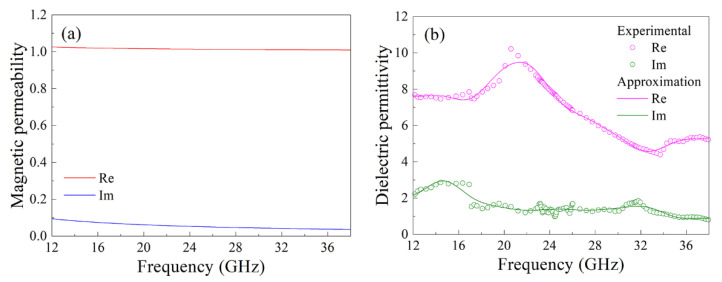
Frequency dependences of magnetic permeability (**a**) and dielectric permittivity (**b**) for the composite with 15% flakes.

The results of calculation of the frequency dependences of the real and imaginary parts of magnetic permeability, for the composite with 15% flakes, carried out following Equation (12) and taking into account the results of approximation of the magnetic permeability of the material of flakes and the following averaging following Equation (15), are shown in Figure 6a.

In Figure 6b, the frequency dependence of the dielectric permittivity of the composite is presented, which is restored from the measurements of the transmission and reflection coefficients. This dependence is obtained according to the method described above with the sliding frequency window. The frequency dependences of the permeability, which are smoothed using the fast wavelet transform-based wavelet filtering algorithm, are shown as solid lines. This algorithm is widely used to process the measurement results of various physical quantities [29,30]. We have used the following parameters of the wavelet filtering here: the wavelet function is sym10 from the symlets family, the number of decomposition levels is equal to 8, and the thresholding strategy is to zero out all the detail coefficients. The mean values of the real and imaginary parts of the dielectric permittivity for three frequency ranges, as well as the values of the microwave conductivity for the composite with 15% flakes, are listed in Table 1. In this table, the values of dielectric permittivity of the epoxy matrix are also presented. It is seen that added metallic particles essentially increase both the real and the imaginary parts of the permittivity. For the composite with 30% flakes in the frequency range from 26 to 38 GHz the mean values are the following: *ε*′ = 38, *ε*″ = 13 and *σ* = 22 S/m.

## 4. Discussion

**Figure 7:**
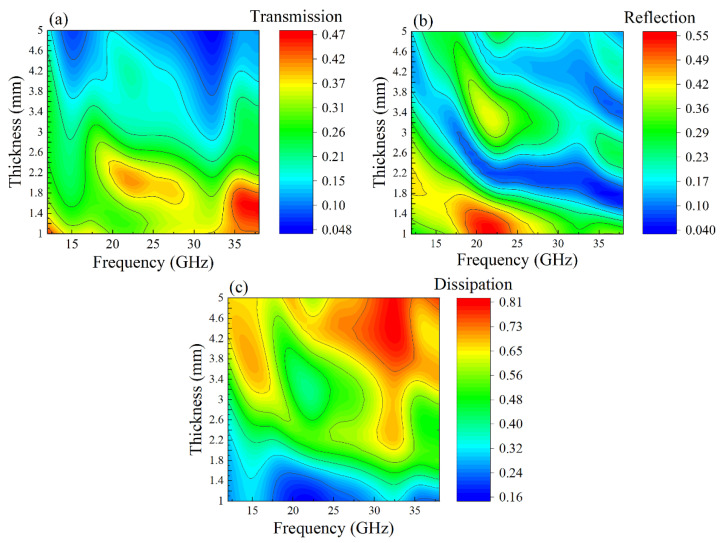
The dependences of transmission coefficient (**a**), reflection coefficient (**b**) and dissipation (**c**) on wave frequency and plate thickness for composite with 15% flakes.

Equations (6)–(8) give the opportunity to calculate the transmission and reflection coefficients and the portion of dissipated power as functions of the wave frequency and the thickness of the composite plate. Of course, the frequency dependence calculated for a fixed thickness equal to the thickness of the sample in the experiment coincides with the experimental dependency. The goal of the calculations is to obtain the frequency and thickness dependences for a wide range of parameters. For more wide applicability of the results of calculations presented below, Equation (4b) for the impedance of the free space is used. The results of calculations of the transmission $T_{P}$ and reflection $R_{P}$ coefficients, as well as the portions of dissipated power D, are presented in graphical form, and the values of the coefficients are represented by colors. In Figure 7, the dependences of transmission (a), reflection (b) coefficients and dissipation (c) on the frequency and the thickness of the plate are shown for the composite with 15% flakes. If one observes these dependences as the functions of either the frequency or the thickness, it is seen that these dependences are nonmonotonic, as a rule. The presence of the extrema is linked with the fact that a quarter or half of the wavelength fits the thickness of the plate.

Because these dependences are nonmonotonic, so, it is reasonable to examine the regions where the coefficients become maximal or, in the opposite, minimal values. Knowing these regions is crucial for applications. Certainly, the transmission coefficient *T_P_* decrease with a substantial increase in the plate thickness, as a whole, owing to absorption. The coefficient *T_P_* assumes the maximal values at frequencies 35–38 GHz when the plate thickness is small and the inequality *λ*_2_ *>> d*_2_ is valid, where *λ*_2_ is the wavelength in the composite. One can see in Figure 7a also two regions of weak transmission for the plates with the thickness exceeding 3–4 mm. These regions are located near the frequencies 15 and 32 GHz. The conducted analysis shows that at frequencies 12–15 GHz, the condition of a quarter-wavelength plate is fulfilled $d_{2}/\lambda_{2}$ = 1/4. Near the frequency of 32 GHz, one wavelength fits the plate thickness. Because the impedances of the plate and the surrounding space differ drastically, therefore the standing wave arises in these frequency ranges. In consequence, the amplitude of oscillations increases, and that is why dissipation grows essentially, see Figure 7c. The module of transmission coefficient is small as a result: $T_{P}$ ≈ 0.05.

The distribution of the regions of small reflection in Figure 7b has a rather complicated view. Among other things, reflection is high in the region *f* = 19–24 GHz but drops down to $R_{P}$ ≈ 0.04 in the same frequency range for thicker plates *d*_2_ ~ 2 mm. The region of small values of reflection in Figure 7b moves to the lower thickness of the plate when frequency increases. Compare Figure 7b,c, one can conclude that the regions of small reflection do not always coincide with the regions of high dissipation. For example, the maximal dissipation realizes for frequencies *f* = 30–34 GHz, where reflection is small but not minimal. Of course, the presence of the local extrema in Figure 7 is caused by interference of forward and backward waves. The frequency-dependent absorption, which is defined by the imaginary parts of dielectric permittivity and magnetic permeability, also influences.

**Figure 8:**
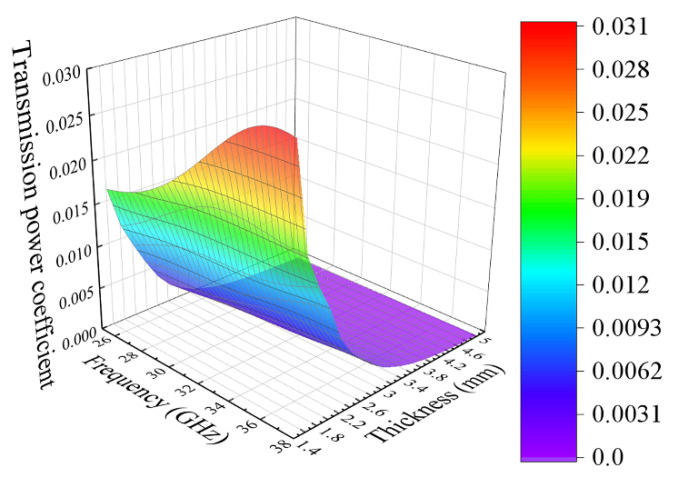
The dependences of the transmission coefficient on wave frequency and plate thickness for the composite with 30% flakes.

Let us now consider the results obtained for the composite with 30% flakes. For this composite, the averaged imaginary part of dielectric permittivity equals *ε*″ = 13, and the microwave conductivity is *σ* = 22 S/m, that is much higher than for the composite with 15% flakes. Therefore, the effect from the interference of forward and backward waves inside the plate has to be essentially lower. In Figure 8, the dependences have been shown of the transmission coefficient on the wave frequency and the thickness of the plate for the composite with 30% flakes. There are no local maximums and minimums in this case. The transmission coefficient decreases rapidly when the thickness arises. Let us perform the analysis of Equations (6) and (7), under strong absorption, when $k_{2}^{\prime \prime }d_{2}>>1$. Given what if x>>1 then the approximate equalities $\sinh\left(x\right)\approx \frac{\exp\left(x\right)}{2}$, $\cosh\left(x\right)\approx \frac{\exp\left(x\right)}{2}$ are valid, we can obtain the following equations:

(16) TP=16Z12|Z˙2|2exp(−k2″d2)4Z12|Z˙2|2+Z14+|Z˙2|4+2Z12Re(Z˙22)+4Z1Re(Z˙2)(Z12+|Z˙2|2)

(17) RP≈Z14+|Z˙2|4−2Z12Re(Z˙22)4Z12|Z˙2|2+Z14+|Z˙2|4+2Z12Re(Z˙22)+4Z1Re(Z˙2)(Z12+|Z˙2|2)

From Equations (16) and (17), the expression can be obtained for the microwave dissipation

(18) D=4Z1[Z1[|Z˙2|2+Re(Z˙22)]+Re(Z˙2)(Z12+|Z˙2|2)]4Z12|Z˙2|2+Z14+|Z˙2|4+2Z12Re(Z˙22)+4Z1Re(Z˙2)(Z12+|Z˙2|2)

It follows from Equation (16) that a monotonic decreasing thickness dependence of the transmission coefficient should exist. Curiously, it is found that, in this case of strong absorption, the reflection coefficient almost does not depend on the thickness of the plate that declares Equation (17).

**Figure 9:**
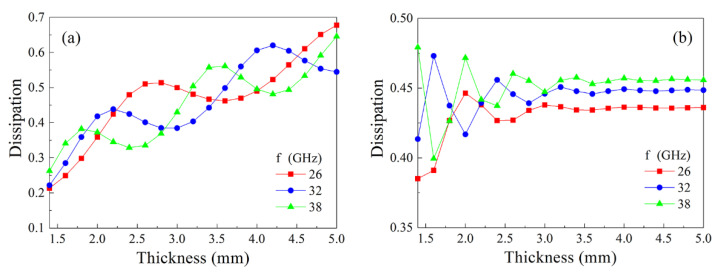
The dependences of microwave dissipation on the thickness of the plate for the composite with 15% (**a**) and 30% (**b**) flakes calculated for several frequencies.

At last, let us discuss the dependence of dissipation on the thickness of the plate. In Figure 9, these dependences are shown drawn for the composites with 15% and 30% flakes at several frequencies. For the composite with 15% flakes, the dependences are oscillating at all frequencies and in the whole range of the plate thicknesses; there is a trend of dissipation increasing while the thickness increases. For the composite with 30% flakes, the oscillations present only for thin plates, so far as the influence of backward waves weakens if the thickness becomes higher. It seems surprising at first glance that the dissipation at high thicknesses of 4–5 mm for the composite with 15% flakes is higher than for the composite with 30% flakes. This peculiarity is caused by the high reflection of waves from the composite with the high content of metallic particles.

## 5. Conclusions

The investigation of the microwave properties of the composites prepared from nanocrystalline flakes of finemet-type alloy has been made. The mean size of flakes is 50 μm. The transmission and reflection coefficients, as well as the portion of dissipated power, have been studied both experimentally and theoretically. The measurements and calculations are carried out in the frequency range from 12 to 38 GHz for the composites with 15% and 30% flakes. 

The method of restoring the frequency dependence of the complex magnetic permeability is worked up, taking into consideration the distribution of spatial orientation of flakes. The frequency dependence of the dielectric permittivity is obtained from the measured frequency dependences of the transmission and reflection coefficients. The frequency dependences of the transmission and reflection coefficients have been calculated for the plate thickness from 1.4 to 5 mm, taking into account the frequency dependences of the magnetic permeability and dielectric permittivity. 

It has been found that the frequency and the thickness dependences of the coefficients are nonmonotonic ones owing to the standing waves. The regions of frequencies and thicknesses where the reflection minimum is observed do not precisely coincide with the maxima of the dissipation. For the composite with 30% flakes, the oscillations in the dependences are weak because of strong absorption.

The obtained results can be useful for the calculations of microwave devices containing magnetic composites.

## Figures and Tables

**Table 1:** Estimations of complex dielectric permittivity and microwave conductivity, averaged over the frequency ranges.

Frequency Range (GHz)	Sample	*ε*′	*ε*″	*σ* (S/m)
12–18	Epoxy matrix	3.22	0.23	
	Composite 15%	7.50	3.13	2.45
18–26	Epoxy matrix	2.94	0.11	
	Composite 15%	8.23	1.52	1.90
26–38	Epoxy matrix	2.61	0.33	
	Composite 15%	5.41	1.10	2.01

## Data Availability

Not applicable.
